# Evaluation of Cystoid Macular Edema Using Optical Coherence Tomography and Fundus Autofluorescence after Uncomplicated Phacoemulsification Surgery

**DOI:** 10.1155/2013/376013

**Published:** 2013-04-30

**Authors:** Muhammed Şahin, Abdullah Kürşat Cingü, Nilüfer Gözüm

**Affiliations:** ^1^Department of Ophthalmology, Faculty of Medicine, Dicle University, 21280 Diyarbakir, Turkey; ^2^Department of Ophthalmology, Faculty of Medicine, Istanbul University, 34093 Istanbul, Turkey

## Abstract

*Aim*. To investigate the utility of fundus autofluorescence (FAF) and optical coherence tomography (OCT) in the evaluation of cystoid macular edema (CME) following cataract surgery. *Materials and Methods*. Forty eyes of 29 patients undergone phacoemulsification, with posterior chamber intraocular lens implantation surgery. Central macular thickness (CMT) of the patients was evaluated using OCT and FAF preoperatively and postoperative 1st, 30th, 60th, 90th, and 180th days. *Results*. CME was detected in three eyes (7.5%) of two patients using OCT. Hyperautofluorescence (HAF) was detected in two of these three eyes and resolved with treatment. In the remaining 37 eyes without CME, there was a significant increase in visual acuity when compared to preoperative values (*P* = 0.008) Mean macular thicknesses (MMT) of the eyes without CME were 174 ± 20 **μ**m preoperatively and 179 ± 22 **μ**m at day 1, 178 ± 19 **μ**m at 1st month, and 168 ± 10 **μ**m at 6th month postoperatively. In the eyes with CME, the MMTs, measured with OCT were 189 ± 23 **μ**m preoperatively and 432 ± 361 on day 1, 343 ± 123 **μ**m at 1st month, 345 ± 196 at 2nd month, and 200 ± 36 **μ**m at 6th month postoperatively. *Conclusion*. We found a moderate increase in CMT in the first 3 months postoperatively, in the eyes without CME which did not cause visual disturbances. FAF is a noninvasive, rapid method for the evaluation and follow-up of CME following cataract surgery.

## 1. Introduction

Cystoid macular edema (CME) is the formation of fluid-filled cystoid spaces between the outer plexiform and inner nuclear layers of the retina, resulting from disruption of the blood-retinal barrier. It is a common complication observed after cataract surgery, with or without other complications. The rate of CME increases in the presence of diabetic retinopathy and uveitis. Although the pathogenesis is still not fully understood, the diagnosis is usually easily confirmed by clinical or angiographic examinations. With modern surgical techniques the incidence of CME has decreased to 1% [1, 2].

The incidence of angiographic CME, without clinical macular edema, has been reported to be around 10–20% following cataract surgery. While it usually occurs 4–12 weeks following surgery; there are a few cases reported after many months or years after the surgery [3].

Optical coherence tomography (OCT) is a noninvasive and quantitative imaging modality, which provides cross-sectional images of the retina, with the help of ~800 nm diode laser light [4–7]. OCT has become an important diagnostic method, especially in retinal diseases, such as CME, diabetic macular oedema, macular hole, and glaucoma.

Autofluorescence (AF) can be defined as light scatter from the structures in the eye, without the use of fluorescein dye. Fundus autofluorescence (FAF) arises from lipofuscin in the retinal pigment epithelium (RPE) cells [8]. Hyper-autofluorescence (HAF) in CME occurs as a result of the displacement of macular pigments into the cystoid gaps. HAF also occurs when inflammation triggers the pro-oxidative pathway. Blue light with a wavelength of 488 nm is used for FAF imaging using Heidelberg Retinal Angiography (HRA) or a modified fundus camera [9, 10].

In the present study, we evaluated the central macular thickness (CMT) using noninvasive methods, including OCT and FAF, in patients who underwent cataract surgery without complication, using phacoemulsification (phaco) with posterior intraocular lens (PCIOL) implantation.

## 2. Materials and Methods

For this study, patients were selected from those who were diagnosed with juvenile or senile cataracts, who underwent phaco and PCIOL implantation with no complications, between October 2008 and June 2009 at the Department of Ophthalmology, Istanbul University, Istanbul Faculty of Medicine. Preoperatively, complete ophthalmologic examinations of the patients were performed, including uncorrected and best corrected visual acuities (BCVA), manifest refraction, keratometry, axial length, intraocular pressure, and biomicroscopic and posterior segment examinations. CMT of each eye was evaluated by a spectral domain-scanning laser ophthalmoscope OCT (SD-SLO/OCT) (OTI, Toronto, Canada). AF images were obtained using a HRA 1 device (Heidelberg Engineering, Germany), using the fluorescein mode without injection of fluorescein and using the red-free mode after the pupils were dilated and focused on the retina. The 30 micron visual field mode was used to obtain FAF images. A series of 20 images were recorded, digitalized, aligned, and averaged using image analysis using HRA 1 device. Patients with any ocular pathology, other than cataracts, were excluded from the study. The study was conducted in accordance with the tenets of the Declaration of Helsinki.

Cataract surgery was performed under local anesthesia, except for two patients whose operations were carried out under general anesthesia. Standard phaco procedures were performed on patients, with a 3 mm clear corneal incision and a foldable PCIOL implantation. Eyes that experienced any intraoperative complication were excluded from the study. Topical prednisolone sodium phosphate 1% (Norsol, Bilim İlaç, Turkey), six times daily, and topical tobramycin 0.3% (Tobrex, ALCON, USA), four times daily, were prescribed to the patients in the postoperative period. Postoperative examinations of the patients were performed on days 1, 30, 60, 90, and 180.

Patients were divided into two groups depending on the presence or absence of CME. An additional examination was performed for patients with CME at 2 months following the operation. Preoperative and postoperative values were recorded and compared between the two groups. Depending on clinical recovery, diclofenac sodium 75 mg tb (Voltaren SR, Novartis, USA), once daily, ketorolac tromethamine 0.5% (Acular, Allergan, Irvine, CA), four times daily, and topical prednisolone acetate 1% (Pred Forte, Allergan, Irvine, CA) were given to the patients with CME who showed no improvement in BCVA.

The findings of the study were analyzed using Statistical Package for Social Sciences (SPSS) for Windows 16.0. the Friedman nonparametric analysis of variance was performed for repeated measurements. The Wilcoxon test was used for comparisons in pairs by applying a Bonferroni correction. Pearson's correlation tests were used to determine the strength of the relationships between the measurements. Significance was evaluated at the level *P* < 0.05.

## 3. Results

Forty eyes of 29 patients were included in this study. Eleven of the 29 patients were male (37.9%), and 18 were female (62.1%). The mean age of the patients was 61.03 ± 16.72 years (range 10–79 years). The mean ages of the patients with CME and the patients without CME were 70.5 ± 10.6 and 60.33 ± 17.01, resepectively. Changes in visual acuity and CMT of patients are shown in Table 1. In the preoperative period, FAF and OCT images of the patients were normal. The foveal examination of the patients with slit lamp biomicroscopy, by using a +90 D noncontact lens, revealed CME in all eyes with the CMT approximately ≥200 *μ*m. Postoperative BCVA did not increase in three of the eyes of two patients (7.5%), and CME was detected in these eyes by the use of OCT, FAF and during routine examination at 1 month.

A significant increase in the BCVA was observed in the eyes without CME after 1 month when compared to the examination at day 1 (*P* = 0.001) and at 3 months when compared to the examination at 1 month (*P* = 0.02), postoperatively (Table 2).

During the followup, there was a significant increase in CMT in the eyes without CME between the preoperative evaluation and day 1 postoperative (*P* = 0.03), at 1 month and 3 months (*P* = 0.05), and a significant decrease in CMT was seen at 6 months, when compared to the examination at 3 months (*P* = 0.04) (Table 3).

The CMT in patients without CME decreased to normal levels at 3 months. However, the preoperative values were lower than the postoperative values measured at 6 months which was not statistically significant. There was no significant correlation between BCVA and CMT in any of the postoperative examinations.

**Figure 1:**
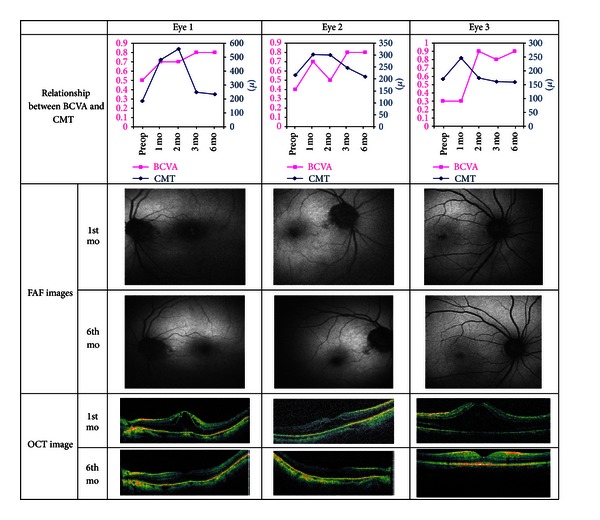
Fundus autofluorescence and optical coherent tomography images and the relationship between the best corrected visual acuity and central macular thickness of the eyes with CME. BCVA: best corrected visual acuity; FAF: fundus autofluorescence; OCT: optic coherence tomography; Preop: preoperative; Postop: postoperative; mo: month.

CME was detected in three of the eyes of two patients at 1 month, but HAF was observed only in two of these three eyes. The change in BCVA and the CMT and FAF and OCT images of these three patients are shown in Figure 1.

## 4. Discussion

CME is the most common cause of unexpected loss of vision after uncomplicated cataract surgery [11]. CME was first described by Irvine after intracapsular cataract extraction (ICCE) in 1953 [12]. In CME following cataract surgery, there are three mechanisms attributing to the etiology of CME: the effect of vitreoretinal traction, light damage, and production of prostaglandins. Development of clinically significant CME, with a decrease in the visual acuity, following modern cataract surgery has been reported at a rate from 0.2% to 14% [2, 13]. Fundus fluorescein angiography (FFA) is the gold standard for the diagnosis of CME. However, as FFA is an invasive and qualitative method, to detect CME without a decrease in visual acuity, there is a tendency to use noninvasive and quantitative methods. OCT and FAF are good, noninvasive, quantitative, and reproducible methods that are used currently.

FFA was compared with OCT by Mitne et al. for the diagnosis of CME, who found an 88% correlation between the two methods [14]. The same comparison was performed by Antcliff et al. who reported that the sensitivity and specificity of OCT was 96% and 100%, respectively [15].

In a study carried out by Perente et al. involving 102 patients who underwent phaco and PCIOL implantation, the CMT (measured using OCT) increased significantly between 1 month and 6 months, postoperatively [16]. In a similar study, Jagow et al. observed a moderate increase in the macular thickness between the 1st week and 6th week, postoperatively, but there was no significant correlation between the CMT and BCVA [17]. Using OCT, Sourdille and Santiago found an association between the increase in the CMT and the decrease in vision after the 1st week postoperative [18].

In the present study, CME has been noticed using OCT in 7.5% of patients after surgery. All of these patients had an unsatisfactory increase in visual acuity. The mean age of the patients with CME was higher than those without CME. This is consistent with publications advocating that aging may predispose the development of CME [19]. Furthermore, the CMT of those patients without CME increased significantly on day 1 following surgery, compared to the preoperative values, using OCT. Light exposure during surgery may explain the increase of the CMT in these patients.

FAF is a new and useful tool for obtaining information on baseline fluorescence from the RPE, as caused by lipofuscin. Lipofuscin mainly accumulates as a result of incomplete destruction of the outer segments of the photoreceptors. A further cause of HAF is inflammation, which triggers the pro-oxidative pathway. FAF is used for diagnosing age-related macular degeneration, hereditary retinal diseases, such as Stargardt disease, retinitis pigmentosa, and cone dystrophies [20, 21].

Displacement of macular pigments due to cystoid gaps in the CME may lead to HAF. Holz et al. stated that extracellular fluid containing retinoid proteins emits autofluorescence. Therefore, in addition to the accumulation of lipofuscin, HAF may be caused by extracellular liquid in the presence of macular edema [9, 10, 22].

FFA and FAF were carried out by McBain et al. in 34 patients suspected of having CME [23]. The sensitivity and specificity of FAF was found to be 81% and 69%, respectively, for the diagnosis of CME. Macaluso et al. and Camparini et al. evaluated patients with CME that developed due to various reasons [24, 25]. They observed a consistency between FAF and FFA in all cases. Similarly, in a further study involving 14 patients, FFA and FAF were found to be correlated with the CME [26].

Peng and Su reported that the correlation between FAF and FFA was 87% in the CME from patients with different origins [27]. Similarly, Pece et al. and Vujosevic et al. reported the HAF as 64.7% and 76.8%, respectively [28, 29].

FAF has some limitations in CME. There is a correlation between the size of the cyst and HAF in CME [8]. Severity of the CME which may be seen as leakage in FA and as increased foveal thickness in OCT also exhibit significant correlation with HAF [28, 29]. So, small-sized cysts with relatively thinner fovea may not be visualized in FAF. HAF in CME is usually apparent under 488 nm excitation while may not be seen under 580 nm excitation [26]. In the current study, one of the eyes with CME could not be detected with FAF image. While we used HRA which has 488 nm excitation, we suggested that this was associated with the small size of the cyst in this eye.

In our study, HAF could not be detected in the eyes without CME. However, HAF was detected in two of the three eyes with CME and was also observed using OCT. The abnormalities in these three eyes were resolved following treatment.

Furthermore, we found that the moderate increase in the CMT in the first 3 months did not cause a decrease in the visual acuity in the patients without CME, and the thickness of the macula gradually decreased and returned to preoperative values within 3 months after surgery. The increased CMT values were regressed in the patients with CME, and HAF also disappeared as a result of the medical treatment, and after 6 months there was no permanent vision loss in any of the eyes.

However, the results of our study are somewhat limited due to the small number of patients and lack of a control group. Nevertheless our results were comparable with the results of the previous studies particularly in demonstration of the relationship between the cyst size and HAF.

In conclusion, in the evaluation of the macula in patients who underwent uncomplicated phaco surgery, HAF was correlated with OCT, demonstrating that HAF can be used as a noninvasive, fast, and convenient method for diagnosis and followup, in the absence of OCT.

## Figures and Tables

**Table 1:** The mean preoperative and postoperative best corrected visual acuity and the mean central macular thickness measurements of patients.

Features	Time of intervention	CME (−) group SD (*n* = 37)	CME (+) group SD (*n* = 3)
	Preop	0.43 (0.21)	0.40 (0.14)
	Postop 1st day	0.51 (0.26)	0.21 (0.14)
BCVA (Snellen)	Postop 1st mo	0.84 (0.21)	0.56 (0.23)
	Postop 2nd mo		0.70 (0.20)
	Postop 3rd mo	0.90 (0.15)	0.70 (0.20)
	Postop 6th mo	0.91 (0.14)	0.83 (0.05)

	Preop	174 (20) *μ*	189 (23) *μ*
	Postop 1st day	179 (22) *μ*	432 (361) *μ*
CMT	Postop 1st mo	178 (19) *μ*	343 (123) *μ*
	Postop 2nd mo		345 (196) *μ*
	Postop 3rd mo	172 (13) *μ*	219 (49) *μ*
	Postop 6th mo	168 (10) *μ*	200 (36) *μ*

CME: cystoid macular edema; SD: standard deviation; Preop: preoperative; BCVA: best corrected visual acuity; CMT: central macular thickness; Postop: postoperative; wk: week; mo: month; *μ*: mikron.

**Table 2:** Statistical significance in the comparison of BVCA measurements without CME.

*P* values	Postop			
	1st day	1st mo	3rd mo	6th mo
Preop	0.26	0.001	0.001	0.008
1st day		0.001	0.001	0.008
1st mo			0.02	0.06
3rd mo				0.18

Preop: preoperative; Postop: postoperative; mo: month.

**Table 3:** Statistical significance in the comparison of OCT measurements without CME.

*P* values	Postop			
	1st day	1st mo	3rd mo	6th mo
Preop	0.03	0.63	0.93	0.79
1st day		0.43	0.24	0.17
1st mo			0.05	0.07
3rd mo				0.04

Preop: preoperative; Postop: postoperative; mo: month.
